# Evaluation of Polyethylene Terephthalate Powder in High Speed Sintering

**DOI:** 10.3390/polym14102095

**Published:** 2022-05-20

**Authors:** Daniel Pezold, Marco Wimmer, Fayez Alfayez, Zahir Bashir, Frank Döpper

**Affiliations:** 1 Chair of Manufacturing and Remanufacturing Technologies, University of Bayreuth, Universitaetsstrasse 30, 95447 Bayreuth, Germany; marco.wimmer@uni-bayreuth.de; 2 SABIC Plastics Application Development Centre, Riyadh Techno Valley Prince Turki Street 1, P.O. Box 5101, Riyadh 11422, Saudi Arabia; fayezaf@sabic.com (F.A.); zbashir2703@gmail.com (Z.B.); 3 Fraunhofer Institute for Manufacturing Engineering and Automation IPA, Universitaetsstrasse 9, 95447 Bayreuth, Germany; frank.doepper@ipa.fraunhofer.de

**Keywords:** additive manufacturing, powder bed fusion, high speed sintering, PET

## Abstract

Laser Sintering (LS) was the first Powder Bed Fusion (PBF) method for polymers and it is now quite an established process for rapid prototyping and even for the production of functional parts. High Speed Sintering (HSS) is a variant of PBF which was later developed and it has the potential to be more scalable than LS. Most of the work for HSS and LS has been conducted with polyamide-12 (PA 12). This work reports the first effort to use polyethylene terephthalate (PET) in HSS. Well defined, simple and complex parts could be printed without any build failures. However, limitations were induced by current HSS machines which led to some curvature (warpage) in tensile bars after manufacturing. The reason for this was that all currently available machines for HSS are built for polymers such as polyamide 12, which means their maximum bed temperature is limited to 190 °C. This corresponds to the lower limit of processability of PET in PBF processes. The slightly curved tensile bars were straightened by heating them to 230 °C with a weight on top, and afterwards the mechanical properties were measured. The tensile modulus was similar to what was obtained with PET via LS but the strength and elongation-at-break (EAB) was lower. Microscopy showed that the reason for the lower strength and EAB was the incomplete melting of particles. This arose from the temperature limitation of the current generation of HSS machines. The porosity was established as 2.23% by helium pycnometry which is the same as for LS. The results of the thermal analysis indicated that the PET parts manufactured with HSS were semi-crystalline like the PET parts manufactured via LS.

## 1. Introduction

There has been a proliferation of ingenious methods of 3D printing, or manufacturing by an Additive Manufacturing (AM) process [1]. Among the methods, Powder Bed Fusion (PBF) is the one with the highest capability of being a truly industrial manufacturing process. The common feature of the PBF process is a bed of powder (polymer, metal or ceramic) which is melted in selected areas, layer by layer, according to the cross-sectional profile of the desired part. Based on the principle of how the energy is input into the powder, PBF for thermoplastic powders can be divided into two sub-categories: Laser Sintering (LS) and High-Speed Sintering (HSS) [1,2]. Laser Sintering, or Selective Laser Sintering (SLS) [3], described in the patent of Beaman and Deckard, is the oldest PBF process, and it is widespread for use in rapid prototyping and industrial manufacturing. Despite the potential of the LS process, it was mainly polyamide 12 (PA 12) that was found to have a wide-enough sintering window. Today, PA 12 has approximately a 90% share of the market, but many amorphous and semi-crystalline polymers are currently used in special niche applications such as polypropylene (PP) copolymers. Although the price of PP resin is about one tenth that of PA 12 resin, the price of LS powders of PP and PA 12 are almost on the same scale. However, PP copolymers have a lower melting point and lower mechanical properties than PA 12. Newer LS powders such as Arnite^®^ T AM 1210 P polybutylene terephthalate (PBT) with a melting point of 225 °C from DSM, and Ultrasint^®^ polyamide 6 with a melting point of 220 °C from BASF, give extended thermomechanical properties than can be achieved with PA 12, but there is not much information yet in the open literature about the use of LS with these thermoplastics.

In 2016, Bashir and Gu (SABIC) filed a patent application describing polyester and related compositions with a specified range of crystallisation half-times, as being suitable for LS [4]. Bashir et al. [5] showed that polyethylene terephthalate (PET) powder had good processibility in LS that was comparable with PA 12. PET also had a wide processing window (like PA 12), but the tensile modulus and strength were higher, and its melting point was ~250 °C compared with 180 °C for PA 12 [5]. Therefore, PET parts should have the potential for end use at a higher temperature than PA 12 parts. Further, heat-exposed PET powder showed stability in molecular weight (unlike PA 12), thus indicating its high re-use potential [6]. Further work by Gu et al. [7] compiled the mechanical properties of PET laser sintered with different orientations and compared it with PA 12. The PET had higher modulus and strength and it showed nearly isotropic properties for the three build directions, but it had a lower elongation-at-break (EAB) and somewhat lower impact resistance than PA 12 [7]. These works also showed the manufactured PET parts were semi-crystalline [5,6,7]. It is difficult to make semi-crystalline parts from PET by injection moulding, hence with PET, PBF delivers something which injection moulding cannot readily do unless oil-heated moulds are used [7].

Hopkinson and Erasenthiran created the HSS method where ink-jet printing was combined with polymer powders for AM [8]. The difference between LS and HSS is as follows. In LS, the cross-sectional layer to be fused is scanned with a moving infrared (IR) laser. The laser spot can move at up to 17.5 m/s in current production machines. The spot size is ~0.18–0.48 mm. Therefore, to cover a large part and/or multiple parts requires a time consuming rastering process [9]. Productivity is increased in some machines by implementing dual lasers for large area builds, but this leads to additional machine cost. In HSS, the cross-sectional area corresponding to the part being manufactured is first printed with an ink using ink-jet print heads in one pass without rastering [8]. The ink is mixed with particles of the radiation absorbing material (RAM) such as carbon black. The powder bed is then scanned by a single pass of a travelling infrared sintering lamp [10,11]. The ink-printed and blackened powder areas absorb the energy significantly better than the unprinted and white powder areas. The printed areas reach temperatures higher than the melting point of the powder material, inducing sintering processes, while the surrounding powder without RAM remains unsintered. A new layer of powder is deposited and the process is repeated. The HSS machine is considered to be cheaper due to its lack of lasers and moving optical systems [10,11]. The university-built prototype HSS machine described by Hopkinson and Erasenthiran was commercialised by voxeljet AG, Germany. Other variants of the HSS method of PBF have been called ‘Multi Jet Fusion’™ (MJF™) from Hewlett Packard and ‘Selective Absorption Fusion’ (SAF™) from Stratasys.

Hopkinson and co-workers researched many aspects of HSS to graduate PBF-based AM from rapid prototyping to rapid manufacturing. They probed various aspects of HSS on part properties such as the amount of ink deposited [12], the effect of build orientation [13] and the effect of bed temperature and infrared lamp power on the mechanical properties of parts [14]. Moreover, the effect of heat on the ink’s viscosity and the possibility of part variation with manufacturing time [15], the spectral emission properties of different infrared lamps [16], thermal gradients in HSS and variation in the flatness of parts made by HSS compared with LS [17,18], and the effect of infrared lamp power and depth of sintering [19] were investigated. Most of the work on HSS was conducted with PA 12 (the same as with LS of thermoplastic powders). However, the feasibility of processing various elastomers [20,21,22,23], polymethyl methacrylate [24] and PP [25] via HSS have also been investigated. Remarkably, Hopkinson’s team also analysed and convincingly showed the economics and scalability of HSS [11].

A current limitation in the PBF of polymer materials is that most of the machines (both LS and HSS) were designed for PA 12. Hence, the maximum bed temperature is limited to 200 °C. Materials such as PBT from DSM (melting point temperature mpt. 225 °C) and PA 6 from BASF (mpt. 220 °C) may be just about processable on LS or HSS machines built for PA 12. The works on LS of PET by Bashir and Gu [4,5], and Gu et al. [6,7] were conducted with an LS machine which could attain the optimal bed temperature required for PET (~230 °C). PET has not been tried for HSS and in fact there is no HSS machine currently that can reach a bed temperature of 230 °C. Despite this, a processing setup could be found in this work to manufacture parts by HSS with build bed temperatures below 200 °C, without machine stoppages and build failures.

## 2. Materials and Methods

### 2.1. The PET Powder

The PET powder used in the previously mentioned work was an experimental grade from SABIC that was used successfully in LS [4,5,6,7]. It had an intrinsic viscosity of 1.122 dL/g measured in 3:2 phenol: 1,2 dichlorobenzene at 25 °C. The particle size analyser Retsch Technology Camsizer XT was used based on dynamic image analysis to obtain the particle size distribution (PSD) curves according to ISO 13322-2. Air pressure dispersion with a dispensing pressure of 70.0 kPa and a gap width of 4.0 mm was set to classify the particles in size categories from 0.0 to 200.0 µm in 2.5 µm steps (volume-based distribution).

The particle shape of the powder was assessed by Scanning Electron Microscopy (SEM). The PET powder sample was prepared by attaching the particles to a 12.5 mm Al SEM stub. The samples were sputter coated with Au-Pd for 2.5 min at a deposition current of 40 mA. The instrument was a FEI Quanta 200 (SEM-2), operating with HV, 20 kV, WD, 15 mm, Spot Size, 3.0 nm, Imaging mode SEI, Image Resolution 1024 × 784.

The melting and cooling curves of virgin PET powder were measured with a Differential Scanning Calorimeter (DSC) to assess the sintering window using a Mettler Toledo calorimeter DSC 1. The powder was heated from 25 °C to 290 °C with a heating rate of 10 °C/min and cooled at the same rate from 290 °C to 25 °C.

The melting of the powder was also observed by hot stage polarizing light microscopy. A small amount of PET powder was placed on a 1.5 cm diameter circular glass slides. The powder particles were covered with a similar glass slides and heated in a hot stage light microscope (Leica DMRXP research light microscope fitted with Linkam THMS600 heating stage) to a temperature of ~280 °C at a heating rate of 10 °C/min. The images were recorded at a magnification of 100× using crossed polarized light mode. This showed the highest temperature up to where the most crystalline, birefringent particles persisted. The flowability and the bridging of melt pools could be observed.

### 2.2. Sieving and Drying Procedure for the PET Powder

PET pellets and powders absorb moisture which can cause a drop in molecular weight due to hydrolysis at melting temperatures. Hence, it is customary and essential to dry PET before conducting any melting process. The virgin PET powder from SABIC was sieved through a Russell Finex Minisifter vibration sieve with a mesh size of 190 µm. The sieved powder was then dried for 5 h at 170 °C inside a Memmert UF55 fan oven. Before filling into the HSS’ powder tank, the dried powder was sieved a second time through a coarse hand sieve with a mesh size of around 1.5 mm. This second sieving step aimed to disperse agglomerates that may have formed while drying which could have interfered with the powder flow.

### 2.3. HSS Machine

**Figure 1:**
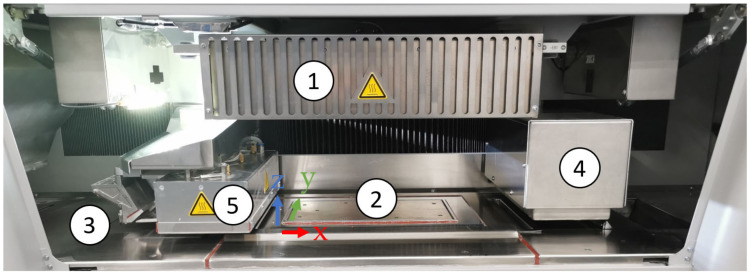
View of the voxeljet VX200 HSS machine: overhead lamp (**1**), built box (**2**), re-coater (**3**), printing module (**4**) and sintering lamp (**5**).

Figure 1 shows the machine view of the inside of a voxeljet VX200 HSS ‘beta version’ that was used to manufacture the HSS PET parts. The overhead lamp (1 in Figure 1) was used to reach the selected process temperature at the powder bed surface. Within, there were six ceramic reflectors facing downwards over the build bed for maintaining a uniform temperature distribution over the powder bed surface. The build box (2 in Figure 1) consisted of four heated side walls and in the vertical z-direction, a vertically moveable and separately heatable floor plate. The dimensions of the build box were 290 mm in x, 140 mm in y, and 180 mm in the z-direction, which resulted in a maximum build volume of 7308 cm^3^.

The stroke of the floor plate defined the thickness of each layer of powder, applied by the heated re-coater (3 in Figure 1) using a vibrating blade system. The powder output was determined by the flow properties of the powder by the re-coater gap and the strength of vibration. Due to the design and vibration of the re-coater system, particle sizes slightly bigger than the layer thickness can be processed.

The ink-printing module (4 in Figure 1) consisted of 3 XAAR 1003 print heads arranged staggered in two rows. These were connected to the machine’s fluid circulation system. The ink used was HSS Ink Type B from voxeljet AG with a carbon-black RAM.

By using a sintering lamp power of 100%, a slow sintering lamp speed of 0.09 m/s and a pre-sintering lamp power of 70% for pre-heating, the built-in cooling system provided insufficient cooling of the sintering lamp. The high power over long periods of time, which was necessary to melt the PET powder, led to errors due to the temperature limitation of the sintering lamp. Therefore, the voxeljet VX200 HSS machine was modified for this work by connecting an additional cooling system to the built-in system for cooling the sintering lamp (5 in Figure 1). Further, thermocouples were installed on the top of the sintering lamp in the print head and in the area of the exhaust air in order to obtain information about the temperature. Both modifications had a negligible impact on the process.

### 2.4. Temperature Recordings with Infrared Camera

Within the VX200 HSS, a built-in infrared camera thermoIMAGER TIM 160S from Micro-Epsilon Messtechnik with a live image as the output was used by the operator for process monitoring purposes. Once per layer (particularly after re-coating new powder and immediately before RAM and IR-radiation of the sintering lamp was applied) a screenshot was automatically taken. Additionally, manually taken screenshots could be saved by the operator to check local temperatures, for instance, when the sintering lamp passed the bed.

### 2.5. Process Conditions

The settings of Table 1 were used for the HSS of PET. As mentioned, the machine was built for polymers such as PA 12 and it could not reach the optimal bed temperature (~230 °C [7]). The temperature hysteresis was set to a maximum of 5 °C which determined the range of acceptable process temperatures. Thereby, the voxeljet VX200 HSS only regulated the overhead lamp power when it exceeded a process temperature of 195 °C or when it fell below 185 °C. By setting the overhead lamp power to ‘high’ for a process temperature overshoot of 195 °C, the machine regulated and reduced the overhead lamp power until 195 °C was reached again. As a result, for a short time and a few layers a higher process temperature of 195–197 °C could be achieved. The build box and re-coater temperatures were set as high as possible without exceeding the limit due to possible fluctuation. The sintering lamp power of 100% was selected in the hopes of achieving the necessary energy to heat the inked areas above the melting temperature. To reduce the curling effects when applying the relatively cold PET powder, the sintering lamp was set to a pre-sintering lamp power of 70%. Therefore, the instant pre-heating of the applied powder reduced the thermal shock and thus the induction of crystallisation of the already printed part underneath [9]. The greyscale in Table 1 describes the amount of ink printed on the powder bed surface. By using a greyscale of level 3, 18 pL of infrared active ink was applied per voxel (volume element).

### 2.6. Layout and Analysis of the PET Parts Manufactured by HSS

**Figure 2:**
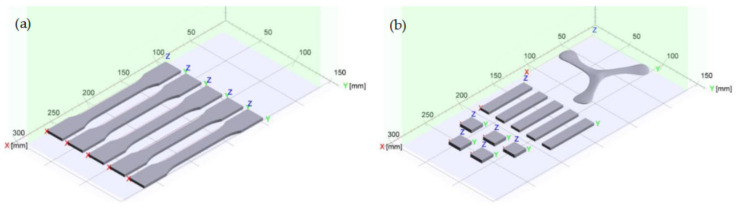
Layout of the build jobs: (**a**) five tensile bars, (**b**) five density cubes, five microscopy cuboids and one three-bladed propeller for demonstration of producibility of complex shapes.

The layouts of the build jobs can be seen in Figure 2. The manufactured parts were five tensile bars (ASTM D638 Type I), five microscopy cuboids (rectangular bars), five density cubes of (17 × 17 × 3.2) mm^3^, and one three-bladed propeller for demonstration of the manufacturing of a complex shape. The height of the build jobs was kept low to reduce the time that the components of the HSS machine were exposed to higher temperatures, in order to avoid damage to the machine.

The density of the cubes was measured using Archimedes’ principle according to DIN EN ISO 1183-1. The weights of the samples were measured with a Kern ALJ 160-4A in air and subsequently within a liquid with a known density. The density $\rho_{S}$ of a sample was calculated with the mass of the sample in air $m_{S,A}$, the mass of the sample in the liquid $m_{S,IL}$, and the density of the liquid $\rho_{IL}$ (Equation (1)). The measurements were performed with water as the liquid at a constant room temperature of 23 °C, for which $\rho_{IL}$ = 0.9976 g/cm^3^.

(1) $$\rho_{S}=\frac{m_{S,A}\times \rho_{IL}}{m_{S,A}-m_{S,IL}}\;\mathrm{in}\;g/{\mathrm{cm}}^{3}$$

Gas pycnometry measurements of virgin powder and a HSS manufactured cuboid were conducted using helium gas and a Micromeritics Accu Pyc II 1340. Each sample was tested ten times. Virgin powder was tested to determine the solid density $\rho_{F}$ of the PET and thus the theoretical maximum density reachable for HSS manufactured parts. The HSS manufactured cuboid revealed the skeleton density $\rho_{S,He}$ which includes the volume of the solid and the closed porosity of the part. Dividing the skeleton density by the solid density allows conclusions to be drawn about the inner porosity $\varepsilon_{i}$ of the PET part (Equation (2)).

(2) $$\varepsilon_{i}=\left(1-\frac{\rho_{S,He}}{\rho_{F}}\right)\times 100\%$$

Tensile tests were carried out to draw conclusions regarding the mechanical properties of the PET parts. Because of a slight upwards curvature occurring in the tensile bars (due to the low bed temperature attainable), they were straightened out by heating in an Memmert UF55 fan oven for 30 min. at 230 °C, with a light aluminium plate on top. Tensile properties were then determined on the straightened bars, testing the five ASTM D638 Type I specimens in a ZwickRoell Z1485 machine with a pre-load of 2.0 N and a testing speed of 5.0 mm/min. Further, five ASTM D638 Type I specimens were manufactured with PA 12 by HSS and tested for mechanical property comparison.

For the manufactured bar, the top and bottom surfaces, and the fracture surface of a tensile bar were investigated by SEM from Thermo Fisher Scientific Inc. ApreoVS using the Everhart-Thornley-Detector (ETD) after sputtering, with a 1.3 nm thick layer of platinum using a Leica EM ACE600.

A DSC melting curve of a manufactured bar was also recorded from 25 °C to 290 °C with a heating rate of 10 °C/min. This gave an immediate indication about whether the PET part manufactured by HSS was semi-crystalline or amorphous. The cooling curve from 290 °C to 25 °C after melting the manufactured HSS part was also recorded to see if the presence of carbon black particles from the ink nucleated the crystallisation of the PET from the melt.

SHORE D hardness measurements were made using a Hildebrand HD3000 Durometer D according to DIN EN ISO 868. Five measurements on each of the top and bottom sides were taken on the shoulder area of an ASTM D638 Type I PET bar and a PA 12 tensile bar. It must be noted that the standard for SHORE D hardness specifies a thickness in the test specimen of 4.0 mm and a distance of 12.0 mm from the measuring point to each edge. The thickness requirement could not be strictly followed as the tensile bar was 3.2 mm thick.

PET is prone to hydrolysis and molecular weight drop (and hence decrease in strength) if moisture is present at melt temperatures. This would cause a decrease in strength and EAB. Any molecular weight drop could be checked by dilute solution viscometry. However, due to the presence of carbon black particles in the HSS part which can interfere with the solution viscometry, the melt volume-flow rate (MVR) was used to compare molecular weight changes (using a Karg Industrietechnik MeltFloW Basic Plus following DIN EN ISO 1133-1 using a temperature of 280 °C and a load of 2.16 kg). The measurements were conducted for virgin powder and a manufactured PET bar. The bar was cut into granules with a size of ~(5 × 5 × 5) mm^3^. Both the virgin powder and the granules from the manufactured specimen were dried for 5 h at 150 °C. The pre-heat time for the polymer to remain inside the heated cylinder was set to 100 s, to allow for melting and temperature equilibration. Within each measurement, 50 values were collected which were used for calculation of the mean value and standard deviation of the MVR.

## 3. Results and Discussion

### 3.1. PET Powder, Particle Size and Particle Morphology

**Figure 3:**
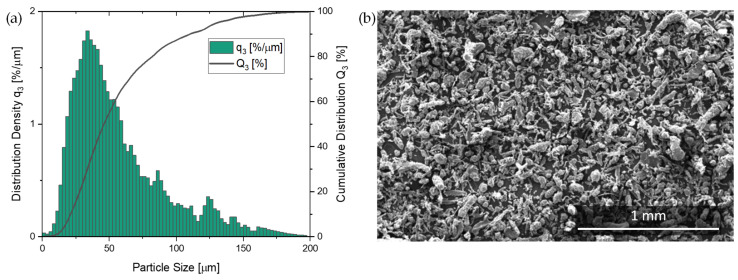
(**a**) Volume-based particle size distribution of sieved and dried PET powder; (**b**) SEM picture of PET powder particles used for HSS.

Goodridge et al. [26] noted that the optimum powder particles for LS should have an average size between ~45.0–90.0 µm and have high sphericity to facilitate flow, reduce the surface area to volume ratio, and improve packing efficiency. Figure 3a shows the volume-based particle size distribution (PSD) after sieving and annealing of the virgin powder. The volume-based PSD in Figure 3a showed a broad distribution (d_10_ of 22.4 µm to d_90_ of 111.6 µm) with a left shifted peak which flattens out towards an increasing particle size of the powder. Powder analysis resulted in a median value d_50_ of 47.3 µm. Figure 3a shows about 15% of the material was >100.0 µm (above the limit indicated by [26]). There was even a small amount of material with a particle size extending to near 200.0 µm.

Further, Figure 3b shows the particle shape of the PET powder which cannot be deduced from the PSD curve in Figure 3a. This was also quite far from the optimal spherical or potato shape [26]. Nevertheless, powder deposition was not an issue and powder flow additives were not needed.

### 3.2. Crystallinity of the PET Powder, the Sintering Window and the Melting Range

**Figure 4:**
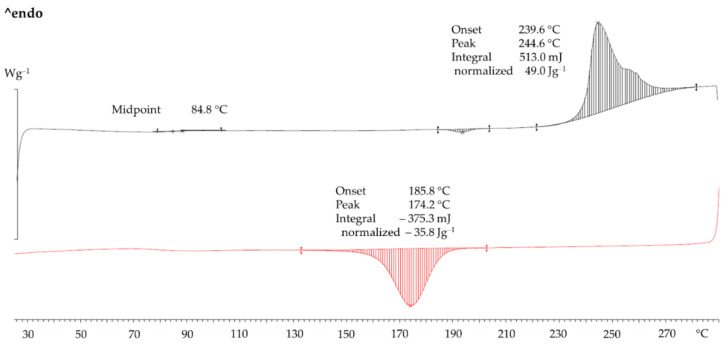
DSC of sieved PET powder and HSS PET bar. Top curve is melting (endothermic) and bottom curve is cooling (exothermic). The clear separation of the melting and crystallisation peaks in the powder is advantageous for PBF for controlling curl.

Figure 4 shows the DSC of the sieved PET powder. In heating (black curve) there was a weak glass temperature *T_g_* with a midpoint value of 85 °C. In fully amorphous PET, at 78 °C (mid-point) there will be a large step-like change due to the big change in heat capacity at the *T_g_*. The heat of fusion was 49 J/g. For substantially crystalline PET samples which do not show cold crystallisation the per cent *X_c_* crystallinity from DSC can be estimated by (Equation (3)):

(3) $$Xc=\frac{\Delta H_{PET\;powder}}{\Delta H_{PET\;single\;crystal}}\times 100\%\;$$

The estimates for Δ*H_PET single crystal_* range between 118 J/g and 155 J/g [27]. Despite the uncertainty in the value for 100% PET single crystal, if one value is selected, relative comparisons can be made. Choosing the value of 118 J/g for Δ*H_PET single crystal_* from Groeninckx et al. [28], *X_c_* for the PET powder was 41.5%.

In Figure 4, on cooling at 10 °C/min after melting at 290 °C, there was a crystallisation peak. The melting and crystallisation curves can be used to assess the sintering window in PBF (54 °C from melting-onset-temperature to crystallisation-onset-temperature difference). The sintering window is even wider than PA 12 [7] and it makes PET tolerant towards processing stress in PBF (LS and HSS).

**Figure 5:**
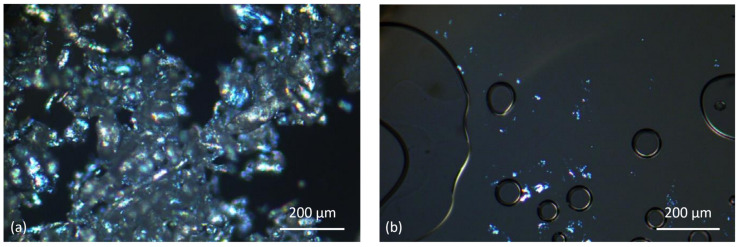
(**a**) Hot stage microscopy of PET powder under crossed polarisers showed softening of particles at 254 °C. (**b**) Polarising hot stage microscopy of PET powder at 272 °C under crossed polarisers, most of the material has melted, coalesced and spread, but there are residual birefringent specks that do not disappear till 280 °C. Circular areas are bubbles with no melt.

Figure 5a,b shows hot stage micrographs of the powder taken between crossed polars. Between 235 °C and 255 °C, softening and slight flow could be seen. Figure 5b shows the melt at 272 °C. While most of the material melted, birefringent specks were still present which only disappeared at 280 °C (not shown). This agrees with the DSC in Figure 4 where the end of melting is between 275–280 °C. Figure 5b also shows the molten powder grains coalesce and the melt spreading easily on glass. Parallel plate viscometry showed that the near-zero shear-rate viscosity of the melt was ~900 Pa s. Low melt viscosity and low surface tension are assets for a good PBF material.

### 3.3. Manufacturing Process and Manufactured Parts

Due to the temperature limitation of the HSS machine it was not possible to operate in the middle or upper end of the sintering window (see Figure 4) as with the LS of PET. Hence, curl could not be avoided. Fortunately, however, it did not completely stop the process. The middle of the sintering window would require a bed temperature of 10–20 °C below the melting peak. In the case of PA 12, the melting peak is at 181 °C, and it is customary to keep the bed at ~170 °C to control curl in the part. This is attainable with all the current HSS type machines. In the case of PET powder with a melting peak of 244 °C (Figure 4), the optimum bed temperature would be 224–234 °C, but this was unattainable (in the current machine). A work-around for this obstacle was used and so the curl was managed such that testable parts could be manufactured. The bed was held at 195 °C (near to the lower edge of the sintering window).

**Figure 6:**
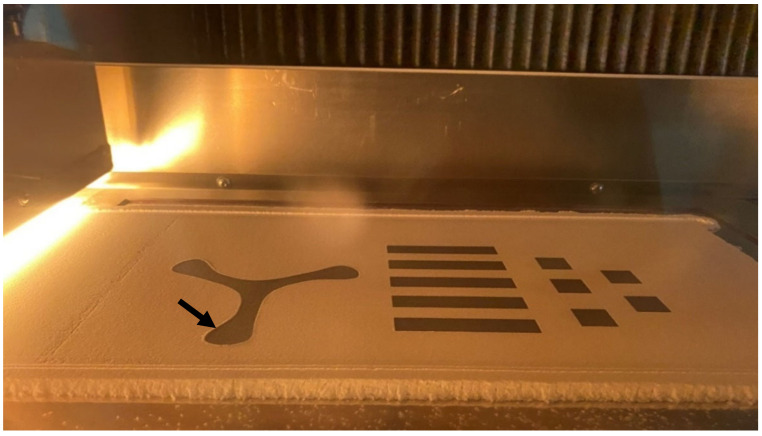
Ink jet printed powder areas are black before the sintering lamp is applied. The blades of the propeller were subject to mild curling (**arrow**).

Figure 6 shows the profile of the parts in one build consisting of rectangular bars, density cubes and a propeller, after ink jet printing with the RAM (carbon black ink) and just before the passage of the travelling sintering lamp. As can be seen here, the blades of the propeller near the build bed walls were subject to curling (arrowed in Figure 6).

Curling occurred when fresh, colder powder was applied on top of the molten area of the first few layers of the part. This rapid cooling of the melt induces crystallization shrinkage (if the polymer is a fast crystallizer) and deformation. Presumably, because the PET crystallises slowly, the curl was not severe enough to cause any builds to fail. This can be contrasted with Williams et al., who found that a good proportion of build failures occurred during HSS with virgin PP powder [25]. In such cases, the edges of the part area curl so heavily and protrude above the bed that the re-coater pulls them out of the powder bed surface and the manufacturing process must be aborted. The normal solution to overcome curl is to increase the bed temperature. Since the temperatures of the build bed were limited to 195 °C and the re-coater to 145 °C, only the increase in the energy input of the sintering lamp was possible. Although the energy input into the entire powder bed was thereby increased, the energy input into the component was also increased. It was assumed that the part area would cool down less during powder application due to the higher temperature caused by the higher input from the sintering lamp, and that the curling effect would be reduced so that manufacturing was not stopped altogether. Indeed, this worked and complete parts could be manufactured with no build failure.

The building time to build a layer was 29 s, so the build time for the tensile bar with 40 layers was 1160 s = 19.3 min (32.0 min with starting and cover layers) and for the rectangular bar with 32 layers, the build time was 928 s = 15.5 min (27.2 min with starting and cover layers).

**Figure 7:**
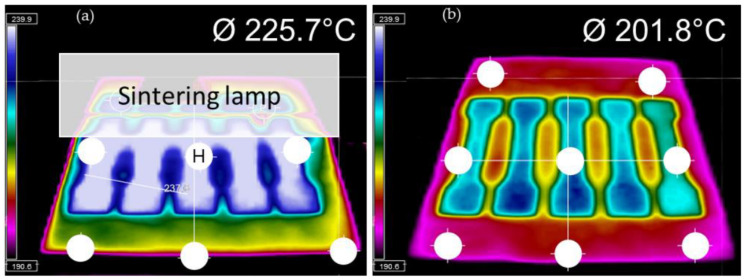
(**a**) Infrared thermal camera images showing the temperature across the bed during the passage of the sintering lamp. Point H was the hottest with a temperature of 244 °C. Note that the sintering lamp cannot be seen as it is made of reflective metal but its position is indicated by the white rectangle and the reflection of the bars is seen. (**b**) Immediately after recoating a new layer of the colder PET powder, the average temperature drops to 201.8 °C.

Figure 7a shows an infrared thermal camera image of the tensile bars during the passage of the sintering lamp over it. The maximum temperature reached at point H in the middle of the central bar in the array was 244 °C. From the DSC in Figure 4 the peak melting temperature of the PET powder was at 244 °C. This means that even at the hottest point in Figure 7b the temperature in the printed areas was just enough to partially melt the PET. Figure 7b shows that when the next layer of powder is added, the average temperature plummeted to ~202 °C. The molten part below cools and crystallises faster than desired, causing shrinkage and curl.

**Figure 8:**
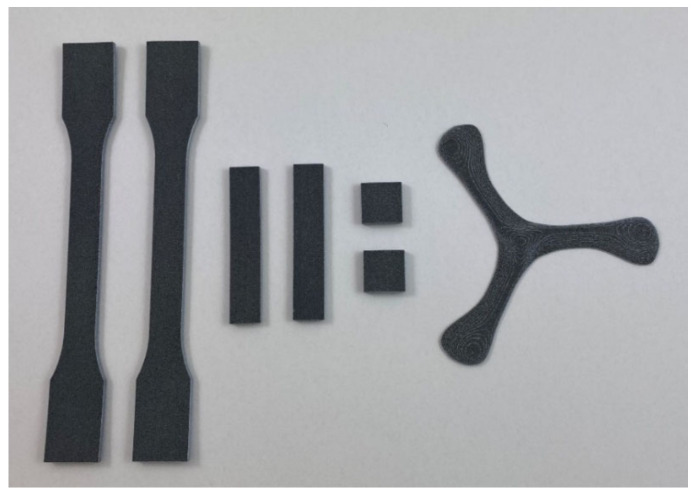
Overview of the different types of parts manufactured from PET by HSS (from left: tensile bars, microscopy cuboids, density cubes and propeller). The propeller showed a pattern of white streaks caused by the energy input of the sintering lamp in combination with a low bed temperature.

Pre- and post-layers of powder are added before and after a build, respectively, for thermal insulation and preserving the powder cake temperature from higher cooling rates of the build bed-floor and overhead-lamp. While manufacturing the specimens, a small curling effect could be seen which disappeared after a few layers (see Figure 6 black arrow). Figure 8 shows the parts extracted from the build bed at the end of the build job, after de-dusting.

However, there was a slight upwards curvature in the bars extracted from the build bed. The curvature is better shown in the view of the tensile bars in Figure 8. This is called warpage and occurred after the actual manufacturing process, during the cooling period before the extraction of the parts from the bed. The warpage of the parts beneath was enhanced by the number of post-layers of powder added after the manufacturing. The colder powder applied by the re-coater on top of the molten specimens without selective energy input led to cooling and faster crystallization than desirable.

**Figure 9:**
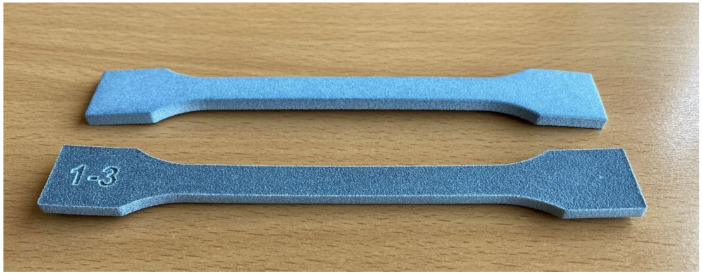
Bottom, warpage of the tensile bars from PET by HSS. Top, an upturned bar to show the bottom surface and the lateral surface are whiter than the top surface which is dark grey. This is due to powder adhesion from outside the build envelope caused by the necessary high energy input through the sintering lamp. The bars could be straightened for tensile measurements by placing them in an oven at 230 °C with an aluminium plate on top.

The top surface of the specimens in Figure 9 appears dark grey due to residuals of carbon black. However, the bottom and the lateral surface appear light grey to white. With PA 12 manufactured by HSS under optimum bed temperatures, all sides of the part appear uniformly grey. For the PET, an energy level of the powder bed is reached, limited by the process temperature of 195 °C. The high and long-lasting selective energy input of the sintering lamp which was necessary to reach sufficient sintering of the first RAM covered powder layer also led to some sintering at the interface towards the surrounding powder particles without RAM. The adhesion of white powder led to a brighter appearance of the lateral and bottom surface of the parts.

The slightly curved tensile bars were straightened by placing them in an oven at 230 °C with an aluminium plate on top. It was possible to heat the bars above the *T_g_* of PET (78 °C) without gross shrinkage and warpage because the manufactured parts from HSS were already semi-crystalline. During 30 min at 230 °C there was no significant further crystallisation. At the same time, semi-crystalline PET softens above 225 °C. Hence, it was possible to obtain straightened bars without loss of shape which could then be used for the tensile measurements.

### 3.4. Crystallinity of the Manufactured Parts by DSC

**Figure 10:**
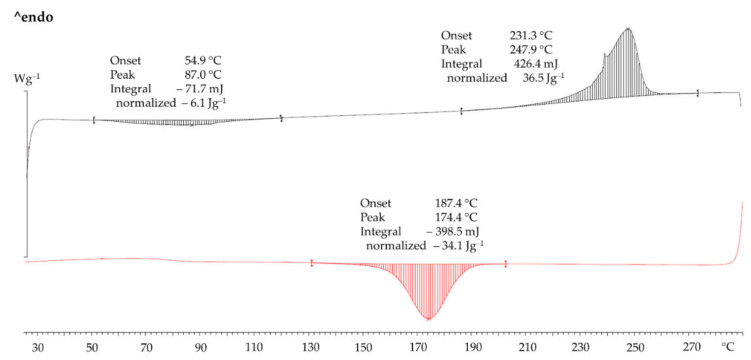
DSC curve of a PET bar manufactured by HSS. The heat of fusion is 36.5 J/g, melting onset is at 231.3 °C, and peak is at 247.9 °C. Note the weak exothermic event with onset at 54.9 °C and peak at 87.0 °C.

The DSC of the manufactured PET part is also shown in Figure 10. Even without X-ray diffraction, from the melting profile in Figure 10 one can deduce that the manufactured PET part is crystalline, just like it is after LS [7]. This is because semi-crystalline PET and amorphous PET have distinctly different DSC melting profiles which are instantly recognisable. In injection moulding with standard cold moulds, the PET parts are amorphous and transparent [7,29]. Amorphous PET has a DSC heating signature which is totally unlike Figure 10 as it shows a prominent *T_g_* at 78 °C followed by a strong cold crystallisation exothermic peak at 130 °C, before the endothermic melting peak *T_m_* [29]. Crystalline parts are preferred as the end use temperature is limited by the *T_m_* rather than the *T_g_*. With PET, amorphous parts are preferred only if transparency is required [7] or if the parts are to be stretched. Hence, the conclusion from the DSC in Figure 10 is that with respect to PET, both PBF processes (LS and HSS) allow semi-crystalline parts to be made, which is more difficult to make by injection moulding. On the other hand, LS and HSS of PET would not allow amorphous parts to be built.

The crystallinity of the manufactured part can be gauged from the heat of fusion in Figure 10 by applying Equation (3) with Δ*H_PET single crystal_* = 118 J/g [28] and Δ*H_PETpart_* = 36.5 J/g Figure 10. Hence, the per cent crystallinity was 30.9% (compared with 41.5% in the virgin PET).

In HSS, there is an additional factor that can narrow the sintering window: the presence of carbon black from the ink in the manufactured part could act as a nucleating agent, promoting faster crystallisation. In a work on LS of mixed powders of aluminium (Al) and PET, Gu et al. [30] reported that the crystallisation peak shifted to higher temperatures because the Al particles acted as nucleating agents for the crystallisation of the PET from the melt [30]. This meant that control of curl was more difficult for Al-PET powders than for PET powder. A similar phenomenon was reported in Al-PA 12 powders [31]. However, Figure 9 shows that on cooling the molten PET part from the HSS manufactured part containing carbon black, the crystallisation onset was at 187 °C and the crystallisation peak was at 174 °C. In the pure powder without carbon black in Figure 4, the crystallisation onset and peak were at 186 °C and 174 °C, respectively. As there was no shift in the crystallisation onset and peak to higher temperatures, the carbon black did not narrow the sintering window. This could be because the amount of carbon black left from the ink is very low whereas in Gu et al.’s work of Al-PET, the Al was present at levels up to 13 vol.%.

Closer inspection of the DSC heating curve of the PET parts (Figure 10) shows a weak and broad exothermic event starting at ~55 °C, which would appear to be below the *T_g_*. This was not seen in the starting PET powder (Figure 4). It can be assumed that this arose from the carrier component of the ink rather than from the carbon black. It is possible that the carrier liquid (petroleum distillates [15]) from the ink acted as a plasticiser lowering the *T_g_* and this caused more of the PET in the part to crystallise during the heating scan (this would be an exothermic event). Examining the DSC curve of a PA 12 part manufactured by HSS, an endothermic event around its *T_g_* could not be observed and it has not been reported in the literature. Further, such a broad and weak exothermic event at 87 °C was not observed in the crystalline PET parts manufactured by LS from the same powder in previous work [7], nor in crystalline PET parts from the same powder by hot powder compaction [32]. Unlike LS, in HSS, besides the solid contaminant (carbon black), the carrier of the ink might leave a liquid residue in the manufactured part. The liquid residue may leave subtle effects on the plasticization of the polymer which could depress the *T_g_*, and the crystallization and melting temperatures of the part. These may occur with some polymers but not others. This aspect appears not to be discussed in the literature.

### 3.5. Density and Porosity

Helium-pycnometry measurements resulted in an average density $\rho_{S,He}$ of 1.3995 g/cm^3^ for the manufactured PET cube and a maximum reachable solid density $\rho_{F}$ of 1.4315 g/cm^3^ for the PET powder. On this basis, a residual inner porosity $\varepsilon_{i}$ of 2.23% of the PET cube was calculated. In the manufactured part, two variables changed and both contributed to lowering the density: (1) the crystallinity of the manufactured part was lower than that of the powder as indicated by DSC (cf. Figure 10 with Figure 4), and (2) the porosity in the article relative to the powder. However, if it is assumed that the lowered density of the part is solely due to voids rather than partially due to lowered crystallinity, then the maximum void content is 2.23%. In fact, it would be somewhat lower as the lower density is partly due to the lowered crystallinity in the part (compared with the crystallinity of the powder).

Similar values for porosity of parts made in LS from similar PET powders was reached by evaluating SEM images [7]. The residual porosity in the manufactured benchmark material, PA 12, was also about 2%, by both LS and HSS.

### 3.6. Mechanical Properties

**Figure 11:**
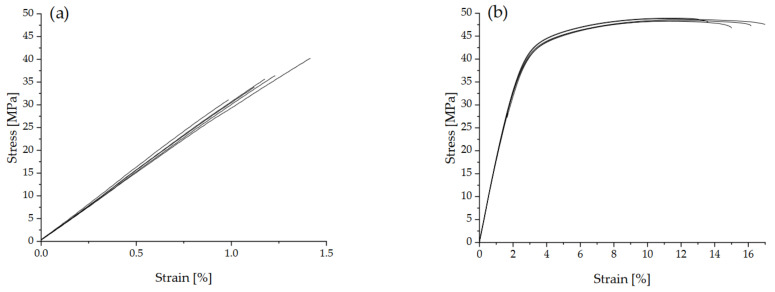
Stress-strain-curves of PET tensile bars manufactured under conditions of the very low bed temperature possible with the HSS machine (**a**) and HSS manufactured PA 12 specimens under optimized conditions (**b**).

Figure 11 shows the stress-strain curves of the PET and PA 12 specimens manufactured with HSS. Both materials showed approximately linear deformation behaviour up to about 35 MPa. While the PA 12 specimens subsequently showed an elastic-plastic behaviour and thus achieved higher EAB, the PET specimens, on the other hand, exhibited brittle fracture behaviour.

Table 2 compares the tensile properties of semi-crystalline PET specimens made by LS and HSS. The values for PA 12 specimens made by HSS are also included. The value for Young’s modulus of the HSS manufactured PET specimens is comparable to its analogue from LS. However, the HSS PET specimens reached only roughly half of the tensile strength of LS ones. Further, EAB was about two to four times lower than in LS. The factors for the brittle behaviour of the PET specimens made by HSS compared with LS could be (1) porosity, (2) a large drop in the molecular weight due to insufficient drying, (3) the straightening step of the HSS PET could have led to post-crystallization and thus to higher Young’s modulus and reduced EAB, and (4) unmelts from insufficient sintering due to the limited temperature. Of these four factors, the porosity would not be the cause for the difference as the value measured for the HSS bars was ~2.2%, and this was the same level as for the PET parts made by LS [7]. The molecular weight drop due to hydrolysis is not likely to be an important cause for the lower strength and EAB. The powder was pre-dried and used without prolonged delay, as in the HSS here, as in the previous LS trials. Further, the infrared camera showed that the highest temperature reached was 244 °C (Figure 7), whereas with the LS, the temperature reached between 270–300 °C. The hydrolysis rate would be lower at lower temperatures. Any major drop in molecular weight that occurred after HSS was checked by measuring the Melt Volume Flow rate of the bar and comparing it with the original powder. This showed that within the experimental variation, there was no difference before and after HSS. The average MVR for the powder was 18.78 g/10 min with a standard deviation of 2.79 g/10 min, and for the part it was 17.92 g/10 min, with a standard deviation 1.56 g/10 min, which was statistically ‘not significantly different’, meaning the molecular weight was unchanged. The possibility of increased crystallization during the straightening operation of the HSS bar (heat to 230 °C for 30 min with a load on top) is also not a major contributing factor. The rate of increase of crystallinity in PET is relatively high if it starts as amorphous, but it levels off after reaching about 30% crystallinity, and thereafter the rise is so slow that it is taken as the ‘ultimate crystallinity’ [33]. The bar from HSS was 29% crystalline, hence its crystallinity would not increase significantly after holding at 230 °C for 30 min during bar straightening. The remaining and dominant factor for the reduced strength and lower EAB is unmelts in the HSS-PET which were much greater in number than in the LS-PET parts [5]. The unmelts are shown in the next section.

For the comparison of LS-PET, against LS-PA 12 (Table 2), a fair set from the previous work has been made—that is, values obtained for optimized LS processes both for PET and PA 12 are compared. The PET made by LS gave about double the modulus of PA 12 made by LS [7]; the tensile strength was 66 MPa for PET vs. 43 MPa for PA 12 [7]; and the EAB was 4.9% for PET (xy build) against 13.1% for PA 12 [7]. The differences between the PET and PA 12 after LS arise from intrinsic material properties. The PET has a more rigid backbone than PA 12 and a higher tensile modulus and tensile strength. Moreover, having a higher *T_g_*, it has an intrinsically lower EAB even when voids are eliminated as occurs with injection moulding.

While the mechanical properties in Table 2 of PA 12 made by HSS are amongst the best achievable and most comparable to others in the literature, the values for PET manufactured by HSS are not the best that can be achieved with this material due to the bed-temperature limitation of the HSS machine (a first attempt with PET).

### 3.7. Surface Morphology, Fracture Surface and Unmelts

**Figure 12:**
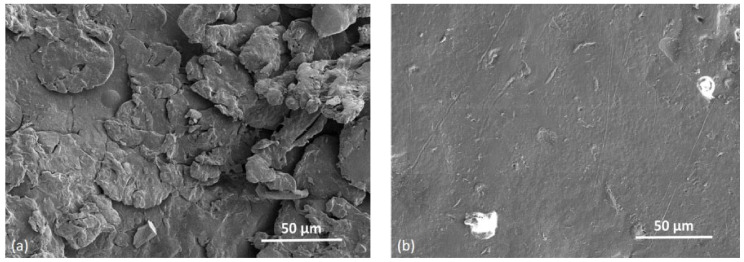
(**a**) SEM picture of the top side of a tensile bar of PET manufactured by HSS with bed temperature of 195 °C, showing unmelts; (**b**) SEM picture of the top side of a PET specimen manufactured from the same powder by LS with the bed temperature at 227 °C, showing fewer unmelts.

Figure 12a shows the SEM picture of the top surface of a PET bar manufactured by HSS with a bed temperature of 195 °C. Unmelts are evident. For comparison, a plate manufactured by LS with the same PET powder with the bed at 227 °C, is shown in Figure 12b. Here, fewer unmelts are evident.

Figure 12a,b support the explanation that the dominant cause for the lower strength of the HSS PET in Table 2, compared with the PET part manufactured by LS, is the lower achievable temperature in our HSS machine, which resulted in more unmelts compared with the LS part where the machine’s bed could be set at 227 °C.

The HSS bars were solid enough for ordinary handling and the unmelts could not be seen by eye. Some of the smaller particles melted and joined together, but the larger ones softened and adhered but did not lose their shape—this is to be expected since the maximum temperature reached in the central bar in Figure 7a was 244 °C, which is lower than the peak of the DSC melting curve of the powder (Figure 4). Further, as the hot stage microscope picture in Figure 5b shows, melting is not fully complete even at 272 °C.

**Figure 13:**
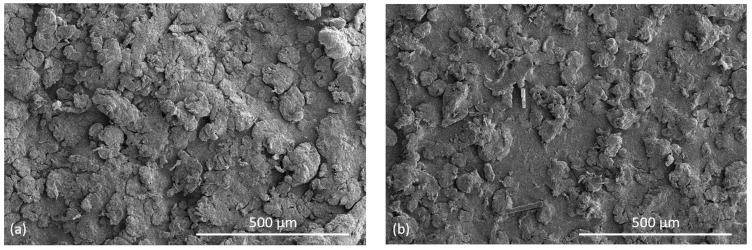
Comparison of top side (**a**) and bottom side (**b**) of a HSS manufactured PET tensile bar.

Figure 13 compares the surface morphology of the top and bottom surface of a tensile bar made from PET by HSS. Both surfaces show unmelts. It can also be seen that there is a difference between the smoothness of the bottom and top surfaces of the manufactured bar. While a flat plane can be seen on the bottom side of the specimen between the semi-molten powder particles that have adhered to it, the top surface of the specimen shows numerous, bigger and inhomogeneous powder particles. Guo et al. [34] as well as O’Connor and Dowling [35] manufactured PA 12 with and without glass beads by MJF (which operates on a similar method as HSS). Both found in the PA 12 and the PA 12 with glass beads that the top surface was rougher than the bottom surface [34,35]. It is assumed that powder particles which are applied on the last layer of the part adhere to the surface. Without the application of RAM and selective energy input, no further sintering is achieved and the surface roughness rises. In contrast, Bashir et al.’s first disclosure of LS of PET [5] showed the opposite. The top surface of the manufactured PET sheet was much smoother than the bottom surface.

**Figure 14:**
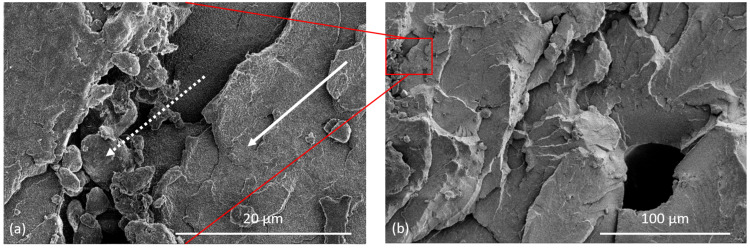
SEM pictures (**a**) of the fractured surface of a tensile specimen showing sintered regions (arrow) and unmelted particles (dotted arrow); (**b**) void comparable in size to the powder particle. The picture in (**a**) is an expansion of the rectangle area in (**b**).

Figure 14 shows the fracture surface of broken tensile bars. Figure 14a reveals that while there are well sintered regions (arrow), there are also unmelted particles present not only on the surface of the bar (Figure 14a,b), but throughout the cross section of the bar (dotted arrow in Figure 14a). In the LS PET parts with the optimum bed temperature of 227 °C, the fractured cross sections show voids but not unmelts [7]. The unmelted particles, like the voids, also act as stress concentrators, lowering the tensile strength of the PET parts. It has been shown [36,37] that unfused or semi-fused particles act as stress concentrators in PA 12 made by LS. The unmelts in our HSS PET were left on the bar’s surface and throughout the cross section of the part because the maximum temperature reached in the bar was 244 °C which was below the DSC melting peak in Figure 4. Further, the PSD curve in Figure 1 shows that there were ~10% particles >100.0 μm, and these melt at a higher temperature. Ibbet et al., showed that if unmelts are closely spaced to each other it would result in a higher probability of failure [38].

Figure 14b shows the fractured surface of a tensile specimen, focusing on a pore with a diameter of about 50 µm. Such voids are also present in LS PET [5,7] and other polymers [39,40] and they lower the strength and impact compared with injection moulded PET. Zhu et al. [39] showed the strength and EAB of HSS PA 12 correlated with porosity: with 5% porosity, the strength was 38 MPa, and EAB was 16%; with 35% porosity, the strength was 12 MPa, and EAB was 5%. However, in the HSS of PET (under the conditions of this work), there are two factors working against strength and EAB: the normal level of void content arising from the PET process, but also a higher number of unmelts.

Table 3 shows the values of the hardness measurements. The SHORE D hardness of the top and bottom side of the PET specimens is each about 10 units higher than of the PA 12 specimens. The top surface of both specimens reached slightly lower values than the bottom side. It is assumed that the rougher and more inhomogeneous top surface compared to the bottom surface is due to the adhesion of powder particles as shown in Figure 13a,b. This reduces the hardness slightly.

### 3.8. Assessment of LS and HSS Machines, and What Is Needed for HSS of PET

According to the cost analysis of Hopkinson and Erasenthiran [1], HSS is not only more cost effective than other AM technologies such as material extrusion and stereolithography, but it can even out-perform LS in terms of cost-per-part. They claim that there are two favourable attributes for HSS over LS: (1) HSS does not use expensive lasers, and this significantly reduces the fixed cost per part and (2) the HSS is estimated to be faster due to sintering all parts in a build in a single pass of the infrared lamp, compared with LS where the laser must be rastered over the selected areas of each part. Although their earlier work [1] claimed that the HSS machine is ~1/3 lower in price than an LS machine due to the absence of a laser and its optics, the current prices of LS and HSS machines do not show such a substantial difference. The price difference between the HSS and LS machines is reduced because several ink-jet print heads are needed in the HSS machine to fit the lateral span of the bed. Nevertheless, the ink-jet print technology enables scalability for the industrial production [2].

However, there are some problems specific to HSS. Majewski et al., noted that the temperature distribution and gradients in a HSS machine are more substantial than in an LS machine [14]. In the HSS machine, there are resistance heaters below the bed, static ceramic lamps above the bed (Figure 1), and repeated traverses of the IR heating lamp over the bed. The heat applied during the travelling sintering lamp’s pass causes a rise in the ambient temperature of the whole machine, including in the ink-jet print heads and associated ducting. Hence, during manufacturing, temperature gradients are set up and they vary with building time which can then lead to differences in the parts that are being built [14,15]. This would be a matter for consistency of product quality and would pose a problem for acceptance as a manufacturing method. With LS, the bed is also heated, but its temperature does not change over the whole area because the energy input area by the laser is smaller. The greater rise in temperature of the bed in HSS (compared with LS) leads to two further issues. Majewski et al. [14] recommended increases (up to a point) in the pre-heat temperature of the powder bed and the infrared lamp power as it includes an increase in the mechanical properties of HSS PA 12 parts. However, the increase in bed temperature and lamp power causes the hardness of the powder cake to increase [14]. In some cases, this occurs to such a degree that it becomes difficult to extricate the part from the powder because the white powder self-sinters. The second issue is a rise in the temperature of the ink. The ink used in HSS is a suspension of carbon black (RAM) in a mixture of petroleum distillates [15]. The ink is continually circulated through the print heads and droplets are delivered to the powder’s surface using a drop-on demand piezoelectric cartridge. Williams et al. [15] investigated the effect of the ink’s temperature during printing on PA 12. This challenge has been surmounted in the voxeljet VX200 HSS machine as an ink temperature regulation system has been implemented. However, it is not clear where the liquid carrier of the ink ends up. Over 200 °C, it evaporates and is expelled by the machine’s venting system, but some small amounts are left in the part. It can change the parts in the case of some polymers (possibly plasticising them and promoting extra crystallisation on heating the part).

HSS manufactured parts introduce contamination from the carbon black of the ink. This would adversely affect the recycling (of the manufactured PET parts) compared with LS manufactured parts. PET is the most recyclable of all polymers, but HSS manufactured PET articles would cause contamination if they were added to the general PET bottle waste, due to the carbon black particles. However, this can be overcome by melt filtration in the PET recycling technology.

With the recent availability of higher temperature materials than PA 12 for LS machines, this works shows the next development needed for HSS machines is the ability of manufacturing with higher build bed temperatures. There will be challenges to consider for designing HSS machines for higher temperature materials, such as the thermal gradients in the bed, and ensuring sufficient cooling of the sintering lamp and the ink.

## 4. Conclusions

Recently, the usability of PET powder was established for LS. PET was characterised by a wide sintering window for LS. As the laser sintered parts were semi-crystalline with a melting point of ~250 °C, PET had useful thermomechanical properties that extended the performance scope beyond what is possible with PA 12 (melting point of 181 °C). Semi-crystalline PET parts are difficult to make by injection moulding but crystalline parts are the normal outcome of PBF.

This work explored the use of PET as a powder for the PBF process of High-Speed Sintering (HSS). HSS is proposed by its developers, Hopkinson and co-workers, as being even more productive than LS due to the cheaper machinery and faster sintering times, as an infrared lamp instead of a scanning laser is used. The results here suggest that in principle PET is equally suitable for HSS. All PET parts in a mixed build could be manufactured without any build failures. Warpage could not be avoided because currently available HSS machines have a maximum bed temperature of 190 °C. Previous LS work with PET powder showed the optimum bed temperature where curl is overcome is 228–233 °C. Due to the bed-temperature limitation of current HSS machines, which were designed for low-melting PA 12, PA 11 etc., the process had to be operated at the lower edge of the sintering window, rather than in the middle. The tensile bars specimens showed some curvature but this will be solvable when a machine with a higher bed temperature becomes available for HSS. For mechanical measurements, the curvature in the PET tensile bars was overcome by heating them to 230 °C with a small weight on top. The modulus was similar (~3.0 GPa) to what was attained with LS of PET and was nearly double that of PA 12. The strength however was 35.5 MPa and therefore lower than that achieved with LS of PET (66 MPa). The reason for this was the incomplete fusion of bigger PET particles, which in turn arose because the bed temperature was below 200 °C. The modulus is a low strain property that was unaffected, but unfused particles showed their effect in lowering the strength and the EAB. Density measurements resulted in a value of residual inner porosity of 2.23% for HSS manufactured parts which is similar to parts from LS. PET specimens that were manufactured by HSS reached a hardness SHORE D of ~79.4 for the top side and ~82.0 for the bottom side, which is about 10 units higher values than comparable PA 12 specimens.

## Figures and Tables

**Table 1:** Process parameters used for manufacturing PET specimens by HSS.

Parameters	Values
Material	SABIC’s experimental PET powder
Powder ratio	100% virgin
Process temperature	190 °C
Temperature hysteresis	5 °C
Build box wall temperature	150 °C
Build box floor plate temperature	185 °C
Re-coater temperature	145 °C
Sintering lamp power	100%
Pre-sintering lamp power	70%
Overhead lamp power	33%
Re-coater speed	0.06 m/s
Sintering lamp traverse speed	0.09 m/s
Ink print head speed	0.39 m/s
Layer thickness	0.1 mm
Re-coater gap	4.0 mm
Vibration strength	87%
Greyscale	3 = 18 pL/voxel
Pre-layers	30
Post-layers	50

**Table 2:** Comparison of mechanical properties of tensile specimens manufactured from PET by LS (Adapted from [7]) and HSS with the same powders. T1 was PET manufactured by LS in a laboratory LS machine with scanning speed of 5 m/s. T2 was PET manufactured by LS in a commercial LS machine with scanning speed of 12 m/s. For HSS, voxeljet Ink Type B at the speed was 0.09 m/s.

	LS			HSS	
Property	PET T1 [7]	PET T2 [7]	PA 12 [7]	PET	PA 12
Young’s modulus [GPa]	2.96	3.26	1.60	3.01 ± 0.09	1.80 ± 0.02
Tensile strength [MPa]	66	64	43	35.5 ± 3.4	48.6 ± 0.3
Elongation-at-break [%]	4.9	2.7	13.1	1.2 ± 0.2	15.0 ± 1.7

**Table 3:** Comparison of hardness SHORE D of tensile specimens manufactured from PET and PA12 in HSS.

Hardness	PET	PA12
Top side [SHORE D]	79.4 ± 0.9	70.8 ± 0.4
Bottom side [SHORE D]	82.0 ± 0.1	72.0 ± 0.7
